# Oxford nanopore technologies—a valuable tool to generate whole-genome sequencing data for *in silico* serotyping and the detection of genetic markers in *Salmonella*

**DOI:** 10.3389/fvets.2023.1178922

**Published:** 2023-06-01

**Authors:** Christine Thomas, Ulrich Methner, Manja Marz, Jörg Linde

**Affiliations:** ^1^Institute of Bacterial Infections and Zoonoses, Federal Research Institute for Animal Health, Friedrich-Loeffler-Institute, Jena, Germany; ^2^RNA Bioinformatics and High-Throughput Analysis, Friedrich Schiller University Jena, Jena, Germany

**Keywords:** *Salmonella*, nanopore sequencing, whole-genome sequencing, *in silico* serotyping, surveillance

## Abstract

Bacteria of the genus *Salmonella* pose a major risk to livestock, the food economy, and public health. *Salmonella* infections are one of the leading causes of food poisoning. The identification of serovars of *Salmonella* achieved by their diverse surface antigens is essential to gain information on their epidemiological context. Traditionally, slide agglutination has been used for serotyping. In recent years, whole-genome sequencing (WGS) followed by *in silico* serotyping has been established as an alternative method for serotyping and the detection of genetic markers for *Salmonella*. Until now, WGS data generated with Illumina sequencing are used to validate *in silico* serotyping methods. Oxford Nanopore Technologies (ONT) opens the possibility to sequence ultra-long reads and has frequently been used for bacterial sequencing. In this study, ONT sequencing data of 28 *Salmonella* strains of different serovars with epidemiological relevance in humans, food, and animals were taken to investigate the performance of the *in silico* serotyping tools SISTR and SeqSero2 compared to traditional slide agglutination tests. Moreover, the detection of genetic markers for resistance against antimicrobial agents, virulence, and plasmids was studied by comparing WGS data based on ONT with WGS data based on Illumina. Based on the ONT data from flow cell version R9.4.1, *in silico* serotyping achieved an accuracy of 96.4 and 92% for the tools SISTR and SeqSero2, respectively. Highly similar sets of genetic markers comparing both sequencing technologies were identified. Taking the ongoing improvement of basecalling and flow cells into account, ONT data can be used for *Salmonella in silico* serotyping and genetic marker detection.

## 1. Introduction

*Salmonella* are food-borne pathogens causing over 1.35 million infections every year in the United States (1). In Europe, 60,050 human salmonellosis cases were registered in 2021 (2). These Gram-negative microorganisms can often be found in the intestinal tract of animals and humans. Different species of farm animals, as well as pets, reptiles, and zoo animals, might serve as reservoirs for *Salmonella*. The pathogen is transmitted to humans mainly *via* contaminated food, water, and by direct contact with infected hosts. The course of infection varies between asymptomatic intestinal colonization and severe systemic disease and is affected by factors of the host and the serovar involved (3).

The genus *Salmonella* comprises two species, namely *Salmonella enterica* and *Salmonella bongori*. The species can be further differentiated into serovars according to differences in their O and H antigen structure. Since serovars of *Salmonella* differ significantly in their virulence, pathogenesis, and host specificity, serotyping plays an important role in the surveillance and controlling of *Salmonella* infections and outbreaks (4).

The traditional method for the determination is serology-based serotyping according to the White–Kauffmann–Le Minor Scheme, which was first published in 1934 (5). Here, the liposaccharide O antigens, as well as the flagellar H1 and H2 antigens, are used in slide agglutination tests to determine the serovar. The O antigens are determined by lipopolysaccharides in the outer membrane of the bacterium. The H antigens may occur in two forms in the flagellum, together or alone, called H1 antigen and H2 antigen. The different variants of antigens are numbered, and the combination of the numbers reflects the antigenic formula of each serovar. The WHO collaborating center for reference and research on *Salmonella* updates regularly the serotyping scheme. Currently, there are over 2600 recognized serovars (6).

The rapid identification of *Salmonella* serovars is essential to gain information on monitoring, epidemiology, and intervention strategies. However, serology-based methods of serotyping are time-intensive and partly limited in their validity (7). *Salmonella* organisms that do not express the O and H antigens in full form, called rough strains, cannot be unequivocally determined by agglutination tests (8). Furthermore, there are strains of different serovars which do not enable the induction of the second H-phase, called monophasic variants. Therefore, the identification of the clear antigenic formula of rough strains and monophasic strains is limited with the traditional slide agglutination test (8).

In recent years, whole-genome sequencing (WGS) of bacteria has been increasingly applied as the costs and time investment of performing WGS and subsequent data analysis have been decreasing (9, 10). WGS can not only reveal information on genotypes of the pathogens but also enable the detection of genetic markers for antimicrobial resistance (AMR), resistance against disinfection agents, plasmids, or virulence, as well as *Salmonella* pathogenicity islands (SPIs) (11, 12). This information can be crucial in outbreak investigations and epidemiological studies and is helpful for effective control strategies. Additionally, WGS has been used for the identification of *Salmonella* serovars and might help overcome the limitations by classical slide agglutination using *Salmonella* antisera (7, 13–16). Tools for serotyping based on WGS data are SISTR developed by Yoshida et al. (14) and SeqSero2 published by Zhang et al. (15).

The tool SISTR analyzes the genetic variations within the O antigens which are encoded by the flippase (*wzx*) and polymerase (*wzy*) genes located in the *rfb* cluster region. To identify the H1 and H2 antigens, the genes *fliC* and *fljB* are analyzed. To achieve the identification, SISTR uses a database based on a *Salmonella* Genoserotyping Array (SGSA) developed earlier by the authors (17, 18). As WGS data can be incorrect due to sequencing errors, SISTR integrates a second step for the serovar prediction. In the first step, the antigenic formula is derived from the four genes representing the antigens which are also considered in the White–Kauffmann–Le Minor scheme. When this antigenic formula is not unique for one serovar due to incorrect or incomplete sequencing data, SISTR uses a specialized multilocus sequence typing scheme (330 loci; for details, see (14)) to identify the serovar in a second step. For this purpose, the phylogenetic clustering of the 330MLST loci is used to determine the single most likely serovar (14).

For SeqSero2, assembled genomes or raw reads can be used to identify *Salmonella* serovars. The tool has two variants. During the variant available for reads and assemblies, the algorithm maps k-mers generated from the input reads/assemblies to a database containing serovar determinants. The other variant is only applicable for read data. Here, all sequencing reads from a query genome were mapped to the serovar determinant database using BWA-MEM (19). Mapped reads are assembled into microassemblies which are then mapped with BLAST again to the database containing the serovar determinants. The mapping for both variants results in the antigenic formula from which the serovar can be inferred (15).

Recently, *in silico* serotyping has been validated with WGS datasets generated with Illumina devices (7, 13–16). Illumina sequencing data are characterized by short, but highly accurate reads. However, given the short reads, genome assembly based on Illumina is complicated, and assembled genomes are rarely completely contiguous. An alternative to this sequencing technique is nanopore sequencing developed by Oxford Nanopore Technologies (ONT). Here, ultra-long reads are generated. The extracted DNA or RNA passes through pores in a membrane. These pores measure the electric current of the electro-resistant membrane. Every nucleotide has a different impact on the current due to their specific nucleobases charge. These differences are afterward translated to nucleotides in a process called basecalling. However, reads from nanopore sequencing with R9.4.1 flow cells have lower accuracy scores compared to Illumina (20). Nevertheless, ONT is constantly improving the flow cells’ performance and basecalling accuracy to achieve similar read accuracy as Illumina sequencing (21).

This study aimed to validate *in silico* serotyping based on WGS data generated with ONT. To this end, *Salmonella* strains of different serovars with epidemiological importance in humans, food, and farm animals (2) were selected to compare results from serotyping using agglutination tests and results from *in silico* serotyping-based sequence data from ONT with a R9.4.1 flow cell as well as Illumina. Moreover, the ability to detect genetic markers and plasmids with ONT and Illumina WGS data was compared.

## 2. Materials and methods

### 2.1. Materials

The dataset, comprised of 28 *Salmonella* strains of different serovars, was selected from the collection of bovine-derived *Salmonella* organisms at the National Reference Laboratory (NRL) for Salmonellosis in cattle in Germany. The NRL receives *Salmonella* organisms isolated at cattle farms in Germany with suspected or confirmed outbreaks of salmonellosis from the investigation offices in the federal states for further characterization. The *Salmonella* organisms selected are of epidemiological relevance and present a wide range of serovars and include strains with special characteristics.

### 2.2. Serotyping

All *Salmonella* strains were serotyped using poly- and monovalent anti-O as well as anti-H sera (SIFIN, Germany) according to the White–Kauffmann–Le Minor scheme (6).

### 2.3. Sequencing and bioinformatics analysis

**Figure 1:**
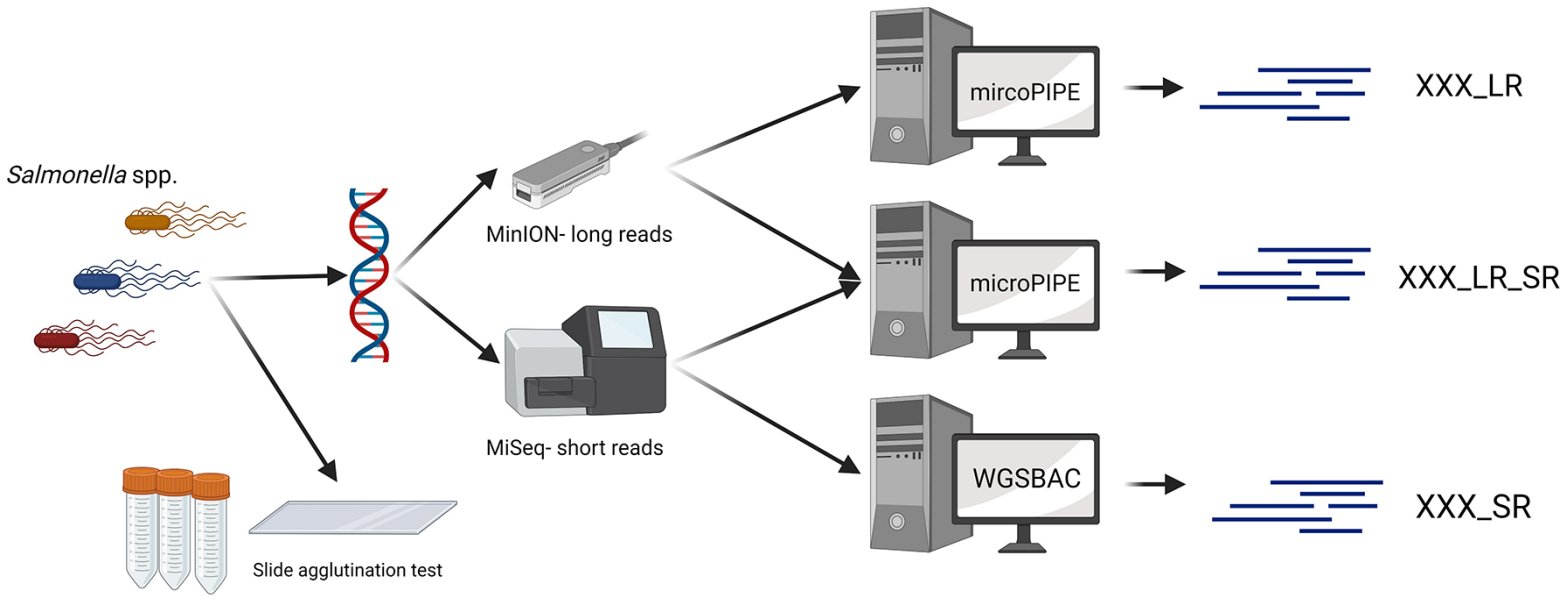
Data generation workflow. For 28 *Salmonella* strains, the serovar was determined with slide agglutination tests. Additionally, all *Salmonella* strains were sequenced with Oxford Nanopore Technologies and Illumina devices. Three different types of assemblies were built (_SR, _LR, _LR_SR). Graphic created with BioRender.com.

All 28 *Salmonella* strains were sequenced with an Illumina MiSeq and a GridION device (Figure 1). Long-read sequencing was performed using a GridION device from ONT. The genomic DNA of all 28 *Salmonella* strains was extracted with the QIAGEN^®^ Genomic-tip 20/G Kit (QIAGEN, Germany) and the Genomic DNA Buffer Set (QIAGEN, Germany). Barcoding was done using the Ligation Sequencing Kit (SQK-LSK109) and the Native Barcoding Expansion Kit (EXP-NBD104). The sequencing was performed with a R9.4.1 flow cell (FLO-MIN106) according to the manufacturer’s instructions.

For Illumina sequencing, the DNA was extracted and purified using the QIAGEN^®^ Genomic-tip 20/G Kit (QIAGEN, Germany) and the Genomic DNA Buffer Set (QIAGEN, Germany). The concentration of the DNA was determined using the Qubit dsDNA BR assay kit (Invitrogen, United States). Sequencing libraries were created using the Nextera XT DNA Library Preparation Kit (Illumina Inc., United States). Paired-end sequencing with a length of 300 bps was performed on an Illumina MiSeq instrument according to the manufacturer’s instructions (Illumina Inc., United States).

If no specific settings are mentioned, the tools utilized for the bioinformatic analysis were used in their respective default settings. Basecalling, trimming, and assembly of ONT data were performed with the pipeline mircoPIPE v 0.9 (22, 23). Guppy v 6.0.1 with the dna_r9.4.1_450bps_hac.cfg model was applied for basecalling and demultiplexing. The quality was checked with NanoPlot v 1.40.2 (24), adapter trimming was performed with Porechop v 0.2.3 (25), and filtering was performed with Japsa v 1.9-10a (26). The FASTQ files containing the trimmed long reads were afterwards assembled using Flye v 2.5 (27). In order to analyze the potential of ONT sequencing, assemblies were built with ONT data only, with ONT data combined with Illumina data and with Illumina data only. For long-read only assemblies (indicated with the ending _LR), the assemblies were only polished with the long reads itself using Racon v 1.4.9 (28) and Medaka v 0.10.0 (29), a polisher from ONT. For the assembly version combining ONT and Illumina data, these polished assemblies were further polished with NextPolish v 1.1.0 (30) and the corresponding short reads to receive the long-read assemblies with short-read polishing (indicated by the ending _LR_SR).

Illumina reads were assembled using the pipeline WGSBAC (v 2.2.0) (31). Here, the coverage was calculated, and the quality was accessed with FastQC (32). Quality and adapter trimming of Illumina reads, as well as, assembly was performed by Shovill v. 1.0.4 (33). These short-read only assemblies are indicated with the ending _SR throughout the study.

All different assembly versions (_LR, _LR_SR, and _SR) were further analyzed with the WGSBAC pipeline. Assembly quality was accessed with QUAST v 5.0.2 (34). Genome annotation was performed with Prokka v 1.13.3 (35), and the assemblies were checked for contamination with Kraken 2 v 0.10.6 (36) and Kraken2DB. The detection of genetic markers for virulence factors was performed with the tool ABRicate v0.8.10 (37) and the Virulence Factor Database (VFDB, version 27 March 2021) (38). Furthermore, AMRFinderPlus (39) was used to detect genetic markers for AMR (genes and mutations) and virulence. For *Salmonella* pathogenicity islands (SPIs), ABRicate together with a previously defined database (FLI, version 14 September 2020) was applied (40). Platon v 1.5.0 was used to detect plasmid-borne contigs (41). *In silico* serotyping was performed with two tools, namely, SISTR (14) and SeqSero2 (15). SeqSero2 was applied with the k-mers assembly-based mode.

## 3. Results

### 3.1. Serotyping

A total of 28 *Salmonella* strains of different serovars were used to test *in silico* serotyping with long-read assemblies compared to slide agglutination tests. Furthermore, short-read-based assemblies were included for comparison. Therefore, the strains were serotyped by slide agglutination (Figure 1 and Table 1). Antigenic formulae of the serovars included in this study are shown in Supplementary Table S2. For three strains, no specific serovar could be determined with the agglutination test. First, no O antigen was detected with the agglutination test for strain 17 PM0072 (formula: O --; H g, p; --). This presumably rough strain resulted in no O antigens and H antigens g and p. For strain 17 PM0296 (formula: O 4, 5; H i; --), the results of the agglutination test indicate a monophasic serovar. The third strain 16PM0121 (formula: O 9, 12; H l, v; --) led to erroneous results presumably due to errors in the synthesis of H2 flagellum.

**Table 1:** Serotyping of 28 different *Salmonella* strains. Comparison of SISTR and SeqSero2 results and long-read only assemblies (_LR) with slide agglutination test results.

Strain	Agglutination test serovar	*in silico* serovar	
		SISTR	SeqSero2
16PM0089	Hadar	✓	✓
16PM0105	Gallinarum	✓	Gallinarum or Enteritidis^a^
16PM0121	O 9, 12 H l,v; -- s^b^	O --; H l,v; --^b^	Goettingen
16PM0122	Abony	✓	4:b:-^b^
16PM0124	III diarizonae	✓	✓
16PM0161	Tennessee	✓	✓
16PM0256	Goldcoast	✓	Goldcoast or Brikama^a^
16PM0296	Anatum	✓	✓
17PM0009	Enteritidis	✓	✓
17PM0296	O 4, 5 H i; --^b^	Typhimurium monophasic	Typhimurium monophasic
18PM0031	Choleraesuis	✓	Paratyphi C or Choleraesuis or Typhisuis^a^
18PM0045	Derby	✓	✓
18PM0092	Paratyphi B	✓^c^	✓
17PM0051	Kentucky	✓	✓
17PM0072	O -- H g, p^b^	Dublin	Dublin
18PM0004	Dublin	✓	✓
18PM0007	Coeln	✓	✓
17PM0282	Typhimurium	✓	✓
18PM0135	Kottbus	✓	✓
16PM0171	Livingstone	✓	✓
16PM0176	Stourbridge	✓	✓
17PM0054	Muenster	✓	✓
17PM0271	Indiana	✓	✓
16PM0056	Bovismorbificans	✓	✓
17PM0024	Mbandaka	✓	✓
17PM0167	Meleagridis	✓	✓
17PM0053	Agona	✓	✓
19PM0148	Infantis	✓	✓

a Correct serovar part of result.

b No serovar predicted.

c SISTR output: Paratyphi B var. Java.

### 3.2. Sequencing quality

DNA from 28 *Salmonella* strains was extracted and genome sequencing was performed with ONT and Illumina. The quality values for each strain and sequencing technique can be found in Supplementary Table S1. Illumina sequencing resulted in an average coverage of 108.86, with a minimum coverage of 30.62 and a maximum coverage of 208.15. ONT sequencing achieved an average coverage of 154.32, ranging from 45.21 to 500.38. The GC content ranged from 51.36% to 52.29%, which is close to the average GC content for the *Salmonella enterica* reference genome (NCBI RefSeq reference genome GCF_000006945.2) with 52% (42). The average read length for the ONT sequencing data was 5408.05, ranging from 2038.6 to 11337.8. For ONT data, the assemblies consist of one to five contigs, whereas the short-read only assemblies (_SR) consist of 26 to 212 contigs with an average of 53.36. Between 68 and 79% of the bases from Illumina sequencing reached Q30 (99.9% accuracy for basecalling). Nearly 6.5% to 15.1% of the ONT sequencing bases reached Q15 quality (96.8% accuracy for basecalling). At least 77.2% (short reads) and 88.96% (long reads) of the raw sequencing reads were classified as belonging to the genus *Salmonella*. The fraction of the reference genome of the short-read assemblies ranged from 67.6 to 98.7% and from 67.9 to 98.1% for the long-read assemblies (Supplementary Table S1).

### 3.3. *In silico* serotyping

*Salmonella* strains of different serovars serotyped by slide agglutination, listed in Table 1, were compared to long-read assemblies with two *in silico* serotyping tools. The results of *in silico* serotyping with short-read-based assemblies are included in Supplementary Table S2.

For ONT data (_LR), SISTR predicted 25 serovars completely matching the results of slide agglutination tests (Table 1). In addition, strain 17PM0072 (agglutination test formula: O --; H g, p; --) was assigned to the serovar *S*. Dublin for ONT data (_LR) and also for short-read-based assemblies (Supplementary Table S2). Strain 17PM0296 was identified as a monophasic variant of *S*. Typhimurium by SISTR. Again, the results of ONT are in accordance with Illumina regarding this strain (Supplementary Table S2). Strain 16PM0121 (through agglutination test O 9, 12 H l, v; --) was not assigned to a unique serovar by SISTR, neither with ONT nor with Illumina data. SISTR reported that only 279 belonging to the MLST330 for the long-read assembly (285 for _LR/_LR_SR) were found (which is below SISTR’s internal quality cutoff of 297). The H1 antigen found by SISTR is not unique for a serovar.

For 3 out of 28 strains, SISTR produced a warning (Supplementary Table S2) when using ONT data (_LR). Warnings were raised for assemblies based on Illumina data (_SR) or the combination of both sequencing technologies (_LR_SR) for two and four strains. Most warnings concerned the amount of cgMLST330 loci identified in the assemblies.

SeqSero2 predicted 21 serovars matching the agglutination test for the long-read only assemblies (Table 1). For the presumably rough strain (agglutination test formula: O --; H g, p; --), SeqSero2 assigned the serovar *S*. Dublin, coinciding with the SISTR results, independent of the sequencing technology (Supplementary Table S2). Similar to SISTR, SeqSero2 identified a monophasic variant of *S*. Typhimurium for strain 17PM0296, independent of the sequencing technology. Three strains resulted in multiple possible serovars when using SeqSero2 (for *S*. Gallinarum, *S*. Goldcoast, and *S*. Cholerasuis). Here, the antigenic formula found by SeqSero2 was not unique for one serovar. In cases where SeqSero2 reported multiple serovars, the serovar identified with the agglutination test was always included in the set of predicted serovars. A message indicated that the serovar should be differentiated with additional tests. Serovar *S*. Abony was not correctly predicted with the long-read assembly. The short-read assemblies resulted in similar false predictions (Supplementary Table S2). For strain 16PM0121, SeqSero2 predicted the serovar *S*. Goettingen, also for the short-read assemblies (Supplementary Table S2).

Overall, *in silico* serovar prediction based on ONT data was highly accurate when compared to the results of traditional slide agglutination. In fact, the tool SISTR achieved an accuracy of 96.4% (Table 2). For the tool SeqSero2, an accuracy of 92% was achieved, not taking results with multiple predicted serovars into account. The non-unique results of SeqSero2 imply further analysis steps, but the set of possible serovars remain significantly smaller with the correct serovar being part of the set. Since all tools independent of the sequencing technology assigned the presumably rough strain as *S*. Dublin, this was considered a correct result. Finally, *in silico* serovar prediction based on ONT data was comparable to results based on Illumina sequencing (Tables 1 and Supplementary Table S2).

**Table 2:** Overall results for SISTR and SeqSero2 for long-read only assemblies (_LR), short-read only assemblies (_SR), and long-read assemblies polished with short reads (_LR_SR).

Serotyping tool	Long-read assembly	Short-read assembly	Long-read assembly polished with short reads
SISTR	96.4% (26 + 1/28)^a^	92.8% (25 + 1/28)^a^	92.8% (25 + 1/28)^a^
SeqSero2	92% (22 + 1/25)^a^^,^^b^	88% (21 + 1/25)^a^^,^^b^	92% (22 + 1/25)^a^^,^^b^

a +1 is the rough mutant form determined as *S. Dublin*.

b The three results being not unique were excluded from the accuracy calculation.

### 3.4. Detection of genetic markers

#### 3.4.1. Virulence factors

To compare the applicability of ONT data to detect genetic markers with the results based on Illumina data, genetic markers for virulence were determined for all assembly types based on short reads only (_SR), long reads only (_LR), and long-read assemblies polished with short reads (_LR_SR). Virulence factors were detected using ABRicate and the Virulence Factor Database (Supplementary Table S3) as well as using AMRFinderPlus (with option --plus). ABRicate reported the same set of virulence factors for 23 of 28 samples (82.1%) when comparing the different assembly types and sequencing technologies. For three strains (*S*. Bovismorbificans, *S*. Meleagridis, and *S*. Typhimurium monophasic), the same set of virulence factors were found by all technologies, but for long-read assembly (_LR and _LR_SR) assembly versions one additional factor. The *S*. Infantis assemblies led to the identification of 116 virulence factors for all assembly types. However, assemblies based on short reads (_SR and _LR_SR) resulted in one additional virulence factors (*entB*). The amount of identical virulence factors was for *S*. Enteritidis 111 (up to six different) (Supplementary Table S3). AMRFinderPlus found genes *iroB* and *iroC* for 27 of 28 strains. *S. enterica* subsp. *diarizonae* (serovar O61; k,1,5,7 (*S*. Ill diarizonae)) was the only strain missing genes *iroB* and *iroC*. For *S*. Typhimurium, *S*. Derby, and *S*. Infantis, the long-read only assembly (_LR) included one gene less than the other two assembly types (missing *iroC, iroC,* and *iroB,* respectively) (Supplementary Table S3).

#### 3.4.2. *Salmonella* pathogenicity islands

Next, the ability of ONT data to detect SPIs was compared to the results from Illumina data (Supplementary Table S4). For 16 strains (57%), the same sets of SPIs were detected by comparing both sequencing technologies (Supplementary Table S4). SPI 1 and SPI 9 were detected in all strains examined with all sequencing technologies. In all strains, except *S*. Abony and *S*. Ill diarizonae, SPI 2 was detected independent of the sequencing technology. The largest number of SPIs was found in *S*. Dublin (rough), *S*. Dublin, and *S*. Typhimurium monophasic (11 for short-read only assemblies and 12 for long-read assemblies). The *S*. Dublin (rough) and *S*. Dublin serovar harbored CS 54, SPI 1, SPI 2, SPI 4, SPI 5, SPI 9, SPI 12–SPI 17, and SPI19. The monophasic variant of *S*. Typhimurium did not contain SPI 17 and SPI 19 but SPI 3 and SPI 6 instead. The lowest number of SPIs was found in *S*. Ill diarizonae with the detection of SPI 1, SPI 18, and SPI 9. The assemblies based on the long-read data (_LR and _LR_SR) led to identical results for the detection of SPIs for all strains. In 12 of 28 cases (43%), the short-read only assembly (_SR) resulted in less SPIs than the long-read-based assemblies. In general, the genomic island CS54 as well as SPI 1–SPI 9 and SPI 11–SPI 19 were found in the WGS data of all *Salmonella* strains.

#### 3.4.3. Plasmids

To compare the ability to detect and assemble plasmids, plasmid-borne contigs were determined with the tool Platon (Tables 3 and Supplementary Table S5). For the short-read only assemblies (_SR), the number of possible plasmid-borne contigs ranged from zero to nine and was larger than for long-read assemblies (zero to four). This is caused by the discontinuousness of assemblies based on short reads (Supplementary Table S1). The long-read-based assemblies (_LR and _LR_SR) led to the same number of plasmid-borne sequences for each serovar. Platon did not find any plasmid-borne contig considering all assembly types for eight *Salmonella* serovars. For additional two strains (*S*. Hadar and *S*. Agona), Platon only characterized contigs of the short-read only assemblies as possible plasmids (7 and 1, respectively).

**Table 3:** The number of plasmids found in 28 *Salmonella* strains. Comparison of Platon results with long-read only assemblies (_LR), short-read only assemblies (_SR), and long-read assemblies polished with short reads afterward (_LR_SR).

Strain	Serovar	Amount of plasmid-borne contigs		
		_SR	_LR	_LR_SR
16PM0089	Hadar	7	0	0
16PM0105	Gallinarum	4	1	1
16PM0121	–	4	2	2
16PM0122	Abony	4	1	1
16PM0124	III diarizonae	1	1	1
16PM0161	Tennessee	1	1	1
16PM0256	Goldcoast	0	0	0
16PM0296	Anatum	0	0	0
17PM0009	Enteritidis	2	1	1
17PM0296	Typhimurium monophasic	5	1	1
18PM0031	Choleraesuis	1	1	1
18PM0045	Derby	0	0	0
18PM0092	Paratyphi B	9	2	2
17PM0051	Kentucky	0	0	0
17PM0072	Dublin (rough)	1	1	1
18PM0004	Dublin	1	1	1
18PM0007	Coeln	0	0	0
17PM0282	Typhimurium	3	1	1
18PM0135	Kottbus	0	0	0
16PM0171	Livingstone	0	0	0
16PM0176	Stourbridge	0	0	0
17PM0054	Muenster	8	4	4
17PM0271	Indiana	0	0	0
16PM0056	Bovismorbificans	1	1	1
17PM0024	Mbandaka	1	1	1
17PM0167	Meleagridis	2	1	1
17PM0053	Agona	1	0	0
19PM0148	Infantis	3	1	1

#### 3.4.4. Antimicrobial resistance

Finally, ONT sequencing and Illumina sequencing were compared regarding the detection of genetic markers for AMR. AMR genes were determined with AMRFinderPlus (Table 4 and Supplementary Table S6). In general, genetic markers for AMR against aminoglycoside, streptomycin, β-lactam, sulfonamide, tetracycline, fosfomycin, streptothricin, trimethoprim, phenicol, chloramphenicol, and quinolone were found across all strains. Assemblies of *S*. Paratyphi B contained the largest number of genetic markers for AMR (resistances against eight antibiotics classes). These include AMR for aminoglycoside/streptomycin, *β*-lactam, tetracycline, quinolone/triclosan, streptothricin, trimethoprim, sulfonamide, and phenicol/chloramphenicol. However, most strains (57.1%) did not result in any hit for an AMR gene with AMRFinderPlus. The different assembly types based on different sequencing technologies yielded the same sets AMR for 25 strain (89.2%). For *S*. Hadar and strain 16PM0121, in the short-read only assembly, additionally, gene *qnrB19* was found compared to assemblies based on long reads. The long-read only assembly (_LR) of *S*. Enteritidis included additionally the *gyr_D87G* gene compared to the other assembly types. For *S*. Tennessee, the long-read only assembly included one gene more (*fosA7*).

**Table 4:** AMR genes found in 28 *Salmonella* strains. Comparison of AMRFinderPlus results with long-read only assemblies (_LR), short-read only assemblies (_SR), and long-read assemblies polished with short reads afterward (_LR_SR).

Strain	Serovar	Antimicrobial resistances^a^		
		_SR	_LR	_LR_SR
16PM0089	Hadar	AG/ST, TET, QUI	AG/ST, TET	AG/ST, TET
16PM0105	Gallinarum	–	–	–
16PM0121	–	AG/ST, SUL, BLA, TRM, QUI	AG/ST, SUL, BLA, TRM	AG/ST, SUL, BLA, TRM
16PM0122	Abony	–	–	–
16PM0124	III diarizonae	–	–	–
16PM0161	Tennessee	–	FOS	–
16PM0256	Goldcoast	–	–	–
16PM0296	Anatum	–	–	–
17PM0009	Enteritidis	–	QUI	–
17PM0296	Typhimurium monophasic	TET, SUL, AG/ST, BLA	TET, SUL, AG/ST, BLA	TET, SUL, AG/ST, BLA
18PM0031	Choleraesuis	–	–	–
18PM0045	Derby	FOS	FOS	FOS
18PM0092	Paratyphi B	AG/ST, BLA, TET, QUI/TCL, STR, TRM, PHE/CHL, SUL	AG/ST, BLA, TET, QUI/TCL, STR, TRM, PHE/CHL, SUL	AG/ST, BLA, TET, QUI/TCL, STR, TRM, PHE/CHL, SUL
17PM0051	Kentucky	–	–	
17PM0072	Dublin (rough)	–	–	–
18PM0004	Dublin	–	–	–
18PM0007	Coeln	–	–	–
17PM0282	Typhimurium	SUL, BLA, TET, PHE/CHL/FFC, AG/ST	SUL, BLA, TET, PHE/CHL/FFC, AG/ST	SUL, BLA, TET, PHE/CHL/FFC, AG/ST
18PM0135	Kottbus	–	–	–
16PM0171	Livingstone	–	–	–
16PM0176	Stourbridge	–	–	–
17PM0054	Muenster	–	–	–
17 PM0271	Indiana	–	–	–
16PM0056	Bovismorbificans	–	–	–
17PM0024	Mbandaka	–	–	–
17 PM0167	Meleagridis	FOS	FOS	FOS
17PM0053	Agona	FOS	FOS	FOS
19PM0148	Infantis	SUL, TET, AG/ST, QUI	SUL, TET, AG/ST, QUI	SUL, TET, AG/ST, QUI

a AG, aminoglycoside; ST, streptomycin; BLA, *β*-lactam; SUL, sulfonamide; TET, tetracycline; PHE, phenicol; CHL, chloramphenicol; FFC, florfenicol; FOS, fosfomycin QUI, quinolone; TCL, triclosan; MAC, macrolide; TRM, trimethoprim; STR, streptothricin.

## 4. Discussion

In this study, sequencing using ONT and Illumina of 28 *Salmonella* strains with serovars of epidemiological and zoonotic relevance was performed (2). Serotyping using two *in silico* tools based on respective genome assemblies was compared to the results from traditional slide agglutination tests. SISTR combined with long-read only assemblies achieved the highest accuracy at 96.4%, and SeqSero2 achieved 92% with the same assembly type. For short-read-based assemblies, SeqSero2 achieved accuracy scores of 92% and 88% accuracy for the _LR_SR version and the _SR version comparable to SISTR (both 92.8%). Considering additionally the three strains, SeqSero2 could not predict uniquely; in this study, SISTR and SeqSero2 did not achieve the same accuracy compared to Uelze et al. (13) when considering short-read assemblies (Supplementary Table S2). The predicted accuracy of 94% of SISTR combined with short-read assemblies as stated by Yoshida et al. (14) and Uelze et al. (13) was slightly improved in this study by the long-read only assemblies.

The slightly better performance of SISTR can be explained by the algorithm SISTR is based on. Both SeqSero2 and SISTR characterize the O and H antigens (14, 15) as an initial step. As the corresponding genes can include sequencing errors, SISTR is performing an additional step: here, a specialized MLST scheme consisting of 330 loci is used to phylogenetically cluster, and the serovar is derived from these clusters (14). Therefore, inconclusive results from the O and H antigen detection can be further identified as a unique serovar with the second step. SeqSero2 is only considering the information derived from the H and O antigens which may lead to non-unique results as seen in this study and previous studies (13, 16).

In this study, SeqSero2 was not able to correctly detect the O antigen of *S*. Abony. Uelze et al. stated that when using the Nextera XT DNA Library Preparation Kit, mapping-based tools such as SeqSero2 struggle with GC-biased sequencing data within the genetic locus of the O antigen, which might result in missing O antigen detection for SeqSero2 (13). Since the Nextera XT DNA Library Preparation Kit was used for sequencing, this result supports these findings. SISTR is not affected by this error source as it combines the identification of O and H antigens with a specialized MLST clustering approach. If the Nextera XT DNA Library Preparation Kit is chosen for sequencing, it is advisable to use SISTR for serotyping to avoid this error.

Strain 21PM0121 was not uniquely identifiable with the slide agglutination test due to the lack of detecting the H2 antigens resulting in the antigenic formula: O 9, 12 H l, v; −. SISTR was not able to identify a serovar. SeqSero2 reported the serovar *S*. Goettingen without any warnings. The H2 antigen reported by SISTR does correlate with the H2 antigen assigned to *S*. Goettingen (in combination with the H1 antigens). The results from the *in silico* serotyping tool indicate that the given strain could be the serovar *S*. Goettingen. This finding indicates an advantage for WGS data *in silico* serotyping compared to traditional agglutination tests as the complete antigenic formula can be determined.

Unlike the agglutination test method, the WGS approaches were able to assign a serovar for a rough strain of *S*. Dublin. Moreover, the two strains of serovar *S*. Dublin resulted in the exact same predictions for AMR and virulence genes as well as SPIs and the number of plasmids, supporting this characterization. Hence, the *in silico* serotyping tool with WGS data has an advantage to the agglutination test method, when considering rough strains of *Salmonella*. Moreover, *S*. Typhimurium and the monophasic variant of *S*. Typhimurium were identifiable and distinguishable with WGS data. This can be difficult with agglutination test methods as the antigens have to be expressed, which is not always the case in every cell condition leading to not decisive results (43). *In silico* serotyping tools are able to report the antigenic formula whereas the agglutination test can be prone to certain error sources (rough form, errors in synthesis, and monophasic variants).

In addition to serotyping, the WGS approach opens the opportunity to perform further bioinformatics analysis to study *Salmonella* strains in detail (44). Especially the detection of genetic markers for virulence and AMR as well as plasmid characterization and SPI detection are important steps in outbreak analysis.

In 23 of 28 (82.1%) *Salmonella* strains, the same virulence factors were determined for the Virulence Factor Database (VFDB) for all assembly types. For three isolates, the long-read assemblies included more factors, which indicates the potential of long-read assemblies for virulence factor prediction. Genes *iroB* and *iroC* were found in 27 of 28 strains, and they belong to the *iro* gene cluster (45). This cluster encodes for a mechanism to evade Lcn2-mediated host defense by mammals, which is blocking the iron uptake of the pathogens (46). The gene cluster is found in various *Salmonella* spp. and helps the organism to undergo this defense mechanism. Strain 21PM0124 is the only serovar of subspecies *S. enterica* subsp. *diarizonae* included in this study. All other strains are serovars belonging to the subspecies *S. enterica* subsp. *enterica*. The specific serovar IIIb 61:k:1,5, (7) is associated with sheep (47, 48). The absence of genes *iroB* and *iroC* in *S. enterica* subsp. *diarizonae* (serovar O61; k,1,5,7) confirms the findings of Uelze et al. (48). The authors indicate that this missing gene cluster could be induced by the adaption to the large intestine of sheep. Here, the pathogen is exposed to a high level of iron and thus does not need the acquisition of iron by salmochelin siderophore (48).

All *Salmonella* genomes encoded SPI 1 and SPI 9 which were identified in various serovars of *S. enterica* and *S. bongori* (49). SPI 1 is crucial for *Salmonella* to invade the epithelial cells of the hosts (50). Apart from genes expressed to help to dock to the host cell, expressed genes belonging to SPI also suppress early proinflammatory cytokine expression of the host’s immune system (50). SPI 9 plays a role in adherence to eukaryotic cells (49). SPI 2 is important for the replication process and the systemic infections within macrophages (51, 52). 21PM0124, where SPI 2 was not detected, is of serovar O61; k,1,5,7. However, genes belonging to SPI 2 (*SlrP, SseF, SseG, SteC, SseJ,* and *SopD2*) were detected within 21PM0124. Opposite to this, other genes belonging to SPI 2 were not detected in the WGS data of strain 21PM0124, e.g., *SifA, PipB2, SteA, SseK1-2, SseL, SspH1-2, SpvB, SpvC,* and *SseL*. This indicates that fractions of SPI 2 are present in sheep-associated serovar but not the complete SPI 2. In general, the observation that short-read only assemblies resulted in lower detection of SPIs (43%) compared to long-read-based assemblies supports the utilization of long-read technologies in the analysis of pathogens. As the detection of genetic markers is performed via sequence alignment to reference databases, the more discontiguous assemblies generated by short reads can lead to a smaller number of hits for those markers compared to assemblies based on ONT data.

For plasmid characterization, differences between data from the two sequencing methods were observed. The applied tool Platon classifies every contig as possible plasmid-borne or chromosomal. The higher amount of plasmid-borne contigs for short-read assemblies is in line with other studies (41, 53). Generally, Illumina-based assemblies contain a higher amount of contigs as the assembly process is more complex due to their short read length. Therefore, plasmids might be separated into multiple contigs using short-read-based assemblies. Here, long-read assemblies have a clear advantage as these reads allow the assembly of the entire plasmid. This explains why the short-read only assemblies in this study contain a larger number of plasmid-borne contigs compared to the long-read-based assemblies. For two strains, *S*. Hadar and *S*. Agona, the long-read-based assemblies (_LR, _LR_SR) resulted in zero plasmid-borne contigs. However, for the short-read only assemblies of those two strains, Platon found seven and one contigs as plasmid-borne contigs. For *S*. Hadar, six of the seven contigs were circular. The plasmid-borne contigs contained Inc. factors, oriT, replication, AMR, and mobilization genes. The only element found on the contig of *S*. Agona was a mobilization gene. As the long-read-based assemblies of these strains consisted only of one contig, no plasmid-born contigs were found. Platon assigns every contig with a size larger than 500 kb as a chromosome (41); therefore, assemblies consisting only of one contig automatically result in zero plasmid-borne contigs.

In this study, only subtle differences between the assembly types and sequencing technologies were observed regarding genetic markers for AMR. One example is missing genes in the long-read only assemblies (*S*. Hadar and O 9, 12 H l,v; −-). Here, the coverage of the gene found in the other two assemblies was identical, indicating that the gene was only part of the short-read data. For *S*. Tennessee, gene *fosA7* […] was only found in the _LR assembly, indicating that it was not covered by the short reads. The frequent appearance of aminoglycoside, tetracycline, sulfonamide, and β-lactam resistance genes was also approved by other studies (54, 55). In addition, the presence of AMR genes of phenicol, chloramphenicol, trimethoprim, quinolone, florfenicol, fosfomycin, and triclonsan was found, in line with other studies (56–62).

For serology-based serotyping, the gold-standard method of the past (6), no expensive equipment is needed (13). However, in theory, all different antisera have to be available in the laboratories for over 2600 identified serovars and must pass strict quality controls to prevent false-positives (13). In addition, the process can be time-consuming and labor-intensive as strains have to express all possible H antigens, involving several analysis steps (7, 13). WGS data not only allow the characterization of O and H antigens but additionally have the utility for the detection of genetic markers for AMR, virulence, and plasmids (63, 64). One advantage of ONT sequencing compared to Illumina sequencing is the generation of ultra-long reads. The length of the reads opens the possibility to create closed assemblies, whereas the short reads of Illumina may lead to discontiguous assemblies (65). Therefore, ONT data allow the assembly of plasmids to closure (66). This can be helpful when analyzing AMR genes as their loci can be studied in more detail. Whether AMR genes are located on plasmids or chromosomes can have an impact on their ability to distribute and is, therefore, an important factor in surveillance (67, 68). Additionally, Illumina sequencing requires relatively high acquisition costs and high costs for sequencing low sample numbers (69). This cost difference can be crucial, especially for small laboratories with lower budgets and small sample numbers. This study shows that *in silico* serotyping and the detection of genetic markers are possible with data based on R9.4.1 flow cells, even though sequencing accuracy is comparably lower (70). Considering that ONT has recently introduced R10.4.1 flow cells with similar sequencing accuracy to Illumina sequencing for DNA (21, 71), ONT sequencing could be an alternative sequencing technique in bacterial genomics. While *in silico* serotyping based on Illumina WGS data is applied on a routine basis in some laboratories (72), ONT sequencing may become more integrated in public health laboratories. This study, in line with other studies in bacterial genomics, shows the potential of ONT sequencing for routine laboratories (73–76). Taking into account the hybrid approach of combining short reads and long reads, laboratories must have both technical devices and trained staff for both sequencing techniques leading to higher costs and more demand on staff. Since the accuracy with ONT alone or Illumina alone is sufficient enough for *in silico* serotyping and the detection of genetic markers, it is advisable to rely on one sequencing technique alone.

In this study, the preciseness of *in silico* serotyping with ONT-generated data was comparable to traditional serotyping. The ongoing development of ONT flow cells, which leads to improved sequencing accuracy, may enable the construction of bacterial genomes with long reads only (21, 71). Moreover, nanopore sequencing has advantages in plasmid characterization due to its simpler assembly process when compared to Illumina sequencing. Additionally, analysis results concerning virulence factors, AMR genes, and SPI detection based on ONT data are comparable to the results from Illumina WGS data. Thus, the results of this study indicate the applicability of ONT sequencing in *Salmonella in silico* serotyping, its ability to lead to more comprehensive surveillance of *Salmonella* outbreaks, and hence its possible use in routine laboratories.

## Data availability statement

The data presented in the study are deposited in the European Nucleotide Archive (ENA) repository, accession number PRJEB59466. Data has been released already and is accessible via https://www.ebi.ac.uk/ena/browser/view/PRJEB59466.

## Author contributions

JL and UM conceived and coordinated the study. CT performed bioinformatics analysis and drafted and wrote the manuscript. UM, JL, and MM critically read the manuscript. All authors contributed to the article and approved the submitted version.

## Conflict of interest

The authors declare that the research was conducted in the absence of any commercial or financial relationships that could be construed as a potential conflict of interest.

## Publisher’s note

All claims expressed in this article are solely those of the authors and do not necessarily represent those of their affiliated organizations, or those of the publisher, the editors and the reviewers. Any product that may be evaluated in this article, or claim that may be made by its manufacturer, is not guaranteed or endorsed by the publisher.

## Supplementary material

SUPPLEMENTARY TABLE S1 Sequencing quality.

SUPPLEMENTARY TABLE S2 Serotyping.

SUPPLEMENTARY TABLE S3 Virulence factors.

SUPPLEMENTARY TABLE S4 Salmonella pathogenicity islands.

SUPPLEMENTARY TABLE S5 Plasmid.

SUPPLEMENTARY TABLE S6 Genetic markers for antimicrobial resistances.
